# Case report: An unusual presentation of renal hypertension after damage control surgery

**DOI:** 10.1016/j.ijscr.2021.105872

**Published:** 2021-04-07

**Authors:** Peter Galos, Michael Hultström, Angeliki Dimopoulou Creusen, Gösta Eggertsen, Jozef Urdzik, Magnus von Seth

**Affiliations:** a Department of Surgical Sciences, Uppsala University, Sweden; b Department of Laboratory Medicine, Karolinska Institutet, Sweden

**Keywords:** Trauma, Hypertension, Page kidney, Renin, Hemorrhage, Hyperreninemia, Case report

## Abstract

**Introduction and importance:**

Hypertensive crisis may be a life-threatening condition to any patient and represents an even more serious condition in trauma patients following severe hemorrhage.

**Case presentation:**

We present a case were surgical drape packing induced hypertensive crisis in a trauma patient, recently resuscitated from abdominal hemorrhage.

**Clinical discussion:**

We argue that direct compression of the kidney by the surgical drapes induced hypersecretion of renin with a mechanism equal to Page kidney. The hypertensive crisis as well as the hyperreninemia was resolved after removing the surgical drapes, and the patient’s condition returned to normal without any sequelae.

**Conclusion:**

We encourage considering this unusual but important complication when packing of the abdomen has been carried out, and strongly recommend ruling out renin-mediated hypertension as a cause of post-operative hypertension in such cases.

## Highlights

• A case of surgical drape packing induced hypertensive crisis in a trauma patient.
• Compression of the kidney from the surgical drapes induced hypersecretion of renin.
• The hypertensive crisis was resolved after removing the surgical drapes.
• Rule out renin-mediated hypertension in hypertension following abdominal packing.

## 1 Introduction

Hypertensive crisis is defined as an arterial blood pressure above 180/120 mmHg and acute end-organ damage [1]. A hypertensive crisis in trauma patients may be particularly dangerous as they increase the susceptibility to end organ-damage such as acute heart failure, respiratory failure and cerebral edema. In addition, high blood pressure may cause re-bleeding from wounds and injuries previously under hemostatic control [2].

Here we present a case from Uppsala University hospital, in whom hypertensive crises appeared after damage control surgery (DCS). We conclude that abdominal packing during DCS caused a direct pressure on the kidney, inducing renal hypertension that was not resolved until the packing material was removed. This is a mechanism equivalent to Page kidney [3,4], which is a rare but well-documented syndrome where subcapsular or perirenal hematomas precipitate renin-dependent hypertension by direct tissue compression [5].

This work has been reported in line with the SCARE criteria [6].

## 2 Case

### 2.1 Day 1 – Trauma and circulatory instability

**Fig. 1:**
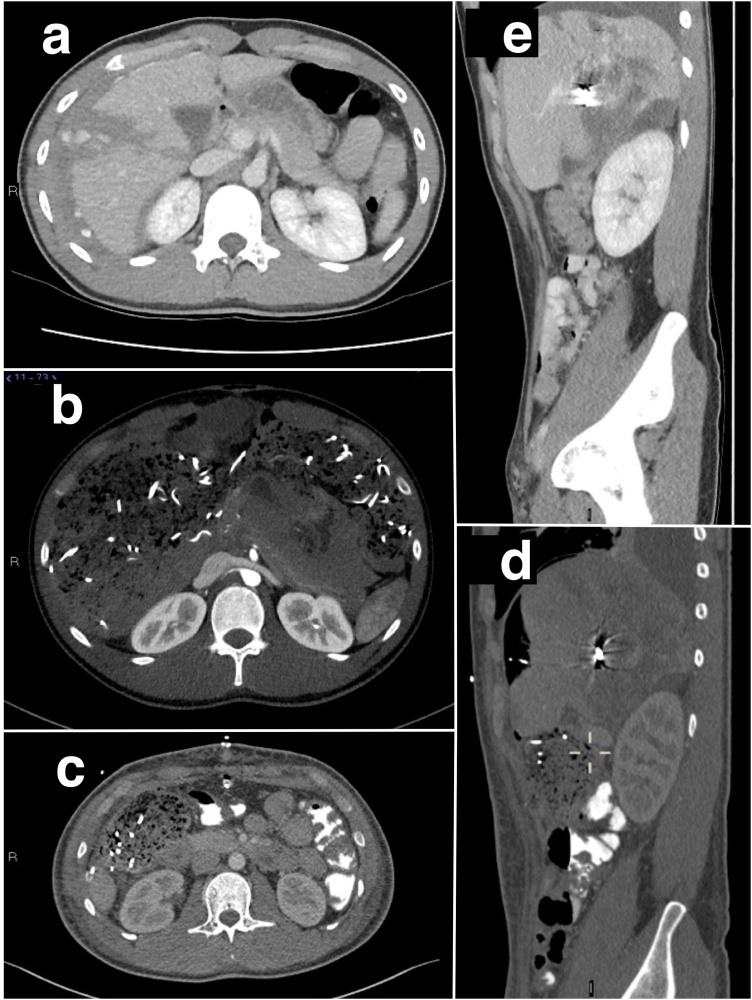
a: Trauma CT of the abdomen demonstrating a complete laceration through the right liver lobe, perihepatic hematoma and ongoing arterial extravasation in the laceration cavity and onwards in the intraperitoneal space. b: Abdominal CT showing the tightly packed abdomen, with visible compression of the retroperitoneal space and drapes directly adjacent to the right kidney. c/d: Abdominal CT showing gauze directly compressing the right kidney indicated by crosshair. e: Abdominal CT approximately ten days post trauma, with visible coils and hepatic lacerations. Right kidney has a normal contour and contrast enhancement.

A 20-year-old man without previous medical conditions suffered blunt abdominal trauma from the handlebar of a motorcycle. Arriving at the local hospital he was in hemorrhagic shock, with computed tomography displaying a liver laceration, capsular tear and free blood in the intraperitoneal cavity (Fig. 1a). The patient was transferred to another hospital where he received massive transfusions and underwent DCS with abdominal packing by 20 pieces of surgical drapes. Postoperatively, he was sedated using ketamine/midazolam and mechanically ventilated.

### 2.2 Day 2 – Development of hypertension

**Fig. 2:**
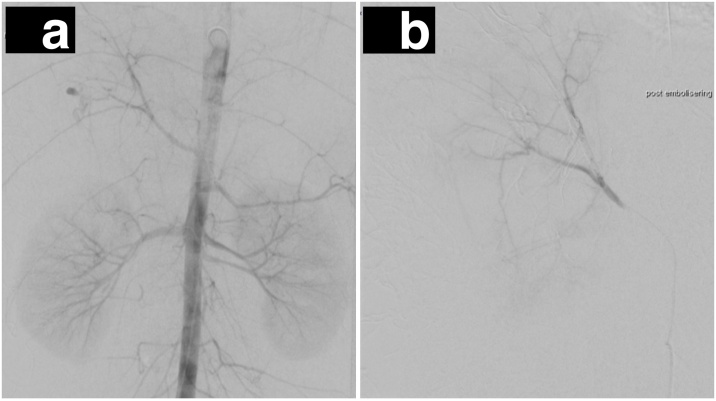
a: Aortic angiogram. Despite the severe vasospasm of the hepatic artery there is an ongoing bleeding at the laceration site. b: Post embolization angiogram of the right hepatic artery, with the bleeding stemmed.

On day 2 he was transferred to Uppsala University Hospital, still dependent on transfusion and vasopressor support. At that time, he had received 4000 ml of Ringers Acetate, 1900 ml of Red cells, 1100 ml fresh frozen plasma, as well as Platelets, Fibrinogen and Tranexamic Acid. Abdominal angiography (Fig. 2a) demonstrated ongoing extravasation from branches of the right hepatic artery, which were selectively micro-catheterized and subsequently embolized (Fig. 2b).

After the angiography he remained circulatory unstable. Computed tomography (Fig. 1b) displayed compression of inferior vena cava as well as congestion including the whole intestine.

When a re-laparotomy was performed, no ongoing bleeding was found and the liver was repacked with two surgical drapes. The patient was now circulatory stable, vasopressor therapy tapered. In the evening, he presented a hypertensive episode with blood pressure rising up to 190/120 mmHg which was thought to be caused by inadequate pain relief.

### 2.3 Day 3 – Treatment resistant hypertension

During the night, antihypertensive treatment was initiated with labetalol, metoprolol and furosemide, and he was extubated in the morning. He negated pain, and at noon a dose of amlodipine was given and Tertiary Trauma Survey (TTS) was performed without any new findings. The blood pressure during the antihypertensive therapy remained around 180/100 mmHg. At this time his cumulative positive fluid balance was estimated to 10 L, but diuretic treatment with furosemide had only a temporary effects on the blood pressure.

### 2.4 Day 4 – Hypertensive crisis

**Fig. 3:**
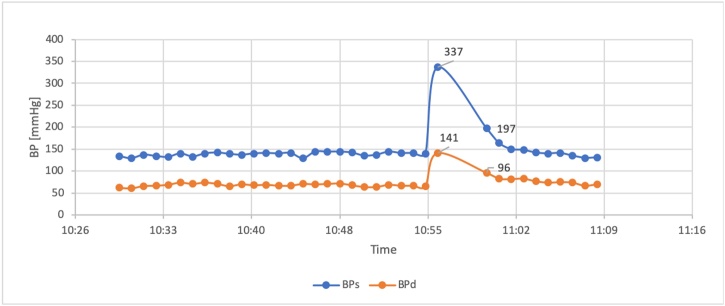
Episode of hypertension registered by PDMS (Patient Data Management System).

On day 4, six episodes of extreme hypertension periods were observed. The highest blood pressure registered by the Patient Data Management System (Fig. 3) was 337/141 mmHg, simultaneous with a heart rate of 93 beats/min and an oxygen saturation of 93 %. The clinical presentation of that event was witnessed at bedside by one of the authors and described as follows:

*“The patient was asleep, with a blood pressure of 160/80 during continuous antihypertensive therapy. Suddenly, a rapid rise in blood pressure was registered and in less than one minute BP rose to 350/200 mmHg. The patient woke up, was pale, and described chest pain, shortness of breath, and showed severe anxiety. Non-invasive blood pressure measurement confirmed the hypertensive crisis. During the episode, saturation dropped to 91 %, while the heart rate did not change. Then blood pressure slowly decreased in* 5 min *down to 160/80 mmHg without additional treatment.”*

The clinical presentation was consistent with a hypertensive crisis, and it was now clear that the patient was in an exceptional, and regarding the recent bleeding trauma, critical condition. Differential diagnoses considered at this time were as follows:

• Cushing Reflex, however, very unlikely due to GCS 15 and no history of neuro trauma
• Insufficient sedation or analgesia
• Renal artery obstruction/dissection
• Pheochromocytoma
• Neuroendocrine pathology including vasopressin, renin or aldosterone
• Transfusion Associated Circulatory Overload (TACO)

The patient was lucid and, except for continuous infusion of clonidine, devoid of sedation. He did not complain of any pain. CT Angiography of the thoracic and abdominal aorta to exclude aortic or arterial dissection were performed without pathological findings, as was Doppler ultrasound of the renal vessels. On CT abdomen, no intra- or extracapsular kidney hematomas could be seen, but a gauze directly compressing the right kidney (Fig. 1c/d) was later found. In order to exclude pheochromocytoma CT images were re-examined by an endocrine surgeon without any positive findings. In addition, plasma samples were drawn for analysis of methoxy-catecholamines, which (later) were found to be normal. Additional treatment with doxazosin and nitroglycerine was started with modest effect on the blood pressure. In addition, all drugs, syringes, and infusion tubes were replaced to rule out accidental drug administration errors.

### 2.5 Day 5 – Page kidney

The suspicion of direct renal compression equivalent to Page Kidney was raised, why plasma renin was analyzed, and found to be 85 mIE/L, thus clearly elevated compared to the reference interval of 2.8–40 mIE/L. At noon, the patient was subjected to re-laparotomy to remove the two remaining surgical drapes. Postoperatively, the hypertension decreased so that during the night following day 5 the patient became normotensive and all antihypertensive therapy could be cancelled. P-Renin decreased to 59 mIE/L during the evening after surgery, and was normalized to 30 mIE/L in the morning of day 6. Creatinine and diuresis were normal during the entire period.

## 3 Outcome and follow up

The patient remained in stable condition after the third laparotomy and was transferred back to the original hospital (Fig. 1e). At a follow-up after 4 months the patient had returned to full-time employment and was feeling well. The blood pressure remained normal without treatment and plasma renin level was found within the reference range (29 mIE/L).

## 4 Discussion including other publications on the subject

Page kidney was first described in 1955 in a patient with subcapsular hematoma [4]. It is considered equivalent to the renal wrap model of renal hypertension developed by Irvine Page and reported in 1939 [7]. This model of renal hypertension will usually progress over several days while the present case developed much more rapidly. It is not possible to exclude that the process was initiated already by the primary surgery and packing. However, the findings are still consistent with packing-induced renal hypertension.

We believe that the hypertension most likely was a renin mediated equivalent to Page kidney, and that the increased levels of plasma renin were caused by DCS packing with direct compression of kidney parenchyma. The clinical presentation was in line with a hypertension mediated by elevated renin levels, generating extremely high blood pressure, flash pulmonary edema, and signs of a hypertensive crisis with acute end organ dysfunction. Importantly, blood pressure and renin levels rapidly decreased after the surgical drape compression was removed. Furthermore, several important differential diagnoses could be excluded, such as drug administration errors, neurotrauma, and renal artery stenosis. Ordinary levels of plasma methoxy-catecholamines usually exclude pheochromocytoma. Modest elevations are, however, observed after treatment with certain drugs such as amlodipine or beta blockers.

For diagnosis of hypertensive cases due to other diseases, i.e. renal artery stenosis and primary aldosteronism, the ratio between aldosterone to renin is preferred to determination of plasma renin concentration, as renin levels without aldosterone are difficult to interpret [8]. However, as the present case shows, isolated renin levels were useful when confirming the diagnosis. The recorded increase in plasma renin is consistent with levels reported in the literature [9].

Differential diagnoses that were, and are, more difficult to exclude are Transfusion Associated Circulatory Overload (TACO) and endogenous catecholamine release. However, non-renin mediated hypertension is expected to display suppressed renin-release, and, in addition, endogenous sympathetic over-activity should cause tachycardia, either of which were not consistent with the presentation in this case. The clinical picture of TACO is not consistent with relapsing hypertensive crises, as in the reported case.

To the best of our knowledge this is the first time that direct renal compression by abdominal packing after DCS is reported to generate a Page kidney with hypertensive crisis in a trauma patient. This is a serious complication to trauma surgery since these patients may be particularly vulnerable to blood pressure increases. In addition, the diagnosis can easily be excluded or verified by analysing plasma renin in conjunction with the discussion of differential diagnosis.

## 5 Take home messages

Although abdominal packing with surgical drapes may be a life-saving procedure in severe abdominal hemorrhage, direct pressure from the drapes could induce secondary insults. In this case, pressure from the packing induced a hypertensive crisis unresponsive to pharmacological therapy but easily resolved by removing the drapes. We encourage considering this unusual but important mechanism when packing the abdomen, and strongly recommend ruling out renin-mediated hypertension in cases who start showing hypertension episodes after surgery.

## Declaration of Competing Interest

The authors state that they have no conflicts of interest for this report.

## Sources of funding

This research did not receive any specific grant from funding agencies in the public, commercial, or not-for-profit sectors.

## Ethical approval

The ethical committee approval was not required given the article type (case report).

## Consent

Written informed consent was obtained from the patient for publication of this case report and accompanying images. A copy of the written consent is available for review by the Editor-in-Chief of this journal on request.

## Author’s contribution

All authors contributed in writing the paper.

## Registration of research studies

Not applicable.

## Guarantor

Peter Galos.

## Provenance and peer review

Not commissioned, externally peer-reviewed.
